# Distribution analysis of structural properties of native porcine humerus–rotator cuff–scapular complex using universal testing machine

**DOI:** 10.1002/jeo2.70164

**Published:** 2025-02-10

**Authors:** Tsuneari Takahashi, Hideyuki Sasanuma, Katsushi Takeshita

**Affiliations:** ^1^ Department of Orthopaedics, School of Medicine Jichi Medical University Shimotsuke Japan

**Keywords:** biomechanical study, rotator cuff, shoulder

## Abstract

**Purpose:**

This study aimed to evaluate the effectiveness of an original mechanical testing setup including the humerus, myotendinous junction and scapula designed to assess the structural properties of rotator cuff attachments in large animals and determine optimal conditions.

**Methods:**

Eight domestic pigs (age, 4–6 months; weight, 30–52 kg) without genetic modifications were euthanized under intubated general anaesthesia control. The scapula–supraspinatus tendon complex with the humerus was excised as a single unit. A device was developed on a universal testing machine to pull in the direction of the tendon's path. After preconditioning at 5 N for 30 s, the composite was stretched to failure at 50 mm/min, and the failure mode was observed. The normality of each structural property was evaluated using the Shapiro–Wilk test. Student's *t* test or the Mann–Whitney U‐test was used to compare whether the structural properties vary in failure modes.

**Results:**

Nine shoulders (56%) ruptured at the humeral attachment; in the remaining seven, the tendon was pulled out from the scapula, leading to rupture. The maximum load and elongation at failure showed normality, whereas the yield load and linear stiffness did not show normality. The mean (standard deviation) maximum load and elongation at failure were 346.4 (99.2) N and 43.1 (12.2) mm, respectively. The median (first and third quartiles) yield load and linear stiffness were 81.2 (63.0, 121.6) N and 15.8 (14.8, 17.5) N/mm, respectively.

**Conclusion:**

This mechanical testing system is useful for evaluating the structural properties of porcine rotator cuff attachments and should be additionally evaluated by cyclic testing.

**Level of Evidence:**

Level IV.

**Abbreviations**
IQR: interquartile range
SD: standard deviation

## INTRODUCTION

In recent years, the use of porcine models for large‐animal research in orthopaedic diseases has increased rapidly. In the field of shoulder surgery, Sasanuma et al. reported that the structure of the attachment of the supraspinatus and infraspinatus tendons to the greater tuberosity of pigs is similar to that of sheep and dogs, and the footprint was large enough to create rotator cuff tear and rotator cuff repair models. They also proposed using the porcine rotator cuff tear model in in vivo studies of shoulder joint diseases in the future [18]. The development of medical materials and devices in tissue engineering and regenerative medicine using large animal models helps improve surgical outcomes. The application of bioengineered materials and autologous and heterologous tendons for the grafted tendons and replacement materials to treat rotator cuff tear in large animal model has been reported [3, 6, 17]. Large‐animal rotator cuff tear models can be used to evaluate the structural properties of the repaired tendons or augmented materials through mechanical testing, cyclic and tensile testing are essential and establishing appropriate testing conditions is crucial. In sheep tendon rupture models, a mechanical testing apparatus is applied at the location of the potted humerus, and the tendon is fixed with a cryoclamp [3, 6, 17, 19]. It is not possible to simultaneously observe both the tendon attachment and the myotendinous junction during cyclic and failure tests in those settings. We have prototyped a mechanical testing device that can be used as an integrated unit of the scapula–tendon–humerus in pigs. This setting may better replicate the human clinical setting, as postoperative changes are seen at both the tendon attachment and the myotendinous junction. We have hypothesized that not only rotator cuff attachment avulsion but also pulling at the rotator cuff–tendon transition was observed during the failure test. Thus, this study aimed to evaluate the structural properties and failure modes of the porcine supraspinatus–infraspinatus–tendon complex using the mechanical testing device in healthy pigs.

## METHODS

All animal experiments were conducted in accordance with the rules and regulations of the Animal Care and Use Committee of Jichi Medical University (Approval no. 21060‐01. Approval date 2022/4/8). Eight domestic pigs (Sanesu Breeding) aged 4–6 months and weighing 30–52 kg without genetic modifications were euthanized by rapid intravenous potassium chloride euthanasia in a deep coma under intubated general anaesthesia control for another anatomical study. Immediately after euthanasia, the scapulothoracic joint was disarticulated from the trunk by experienced Japanese board‐certified orthopaedic surgeons (T. T. and H. S.), soft tissues other than the rotator cuff (supraspinatus–supraspinatus muscle complex) were resected and the humerus was potted in aluminium tubes with cement to create the humerus–tendon– scapular complex in order to avoid changes in structural properties due to rigour mortis [12].

### Biomechanical evaluations

Throughout the procedure, the specimens were kept moist by saline spray. The axial translation of the complex was measured under a drawer force based on previously reported testing conditions [7, 10].

**Figure 1:**
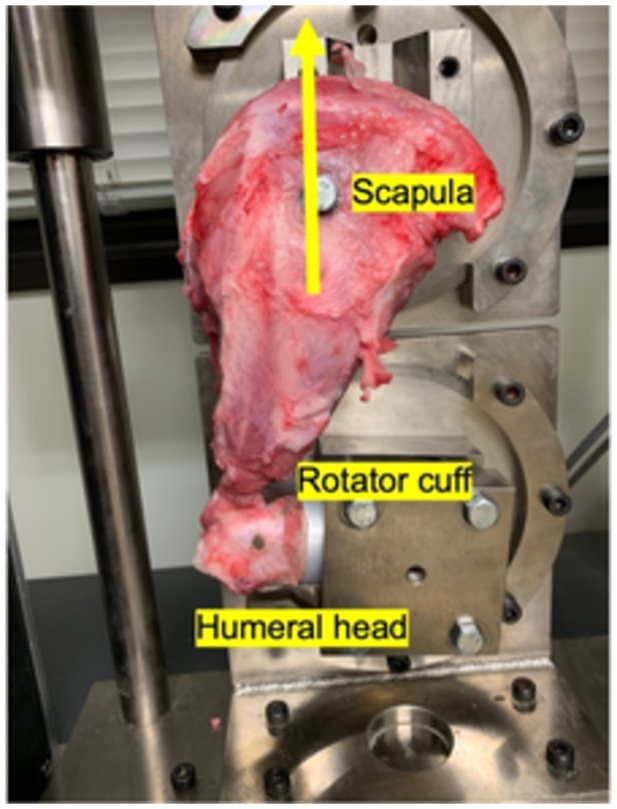
Scapula and potted humerus were fixed to a universal testing machine so that the load was applied horizontally to the orientation of the rotator cuff. After preconditioning, the composite was stretched to failure. A yellow arrow indicates the direction of the traction force.

The scapula and the potted humerus were fixed to a universal testing machine (Tensilon RTG 1250; Orientec) with a set of specially designed grips and screw to hold the scapula to the jig, equipped with a load cell with a maximum load of 2000 N and load measurement accuracy of plus or minus 1.0%, and the traction force was applied horizontally in the direction of the rotator cuff. After preconditioning at 5 N for 30 s, the composite was stretched to failure at a rate of 50 mm/min and observed whether the rupture was due to detachment at the rotator cuff attachment or to pulling at the rotator cuff‐tendon transition (Figure 1). In addition, Tensilon Advanced Controller for Testing (Orientic Co., Ltd.) was used to calculate structural properties (yield load, maximum load, linear stiffness and elongation at failure) [4, 8].

### Statistical analysis

**Figure 2:**
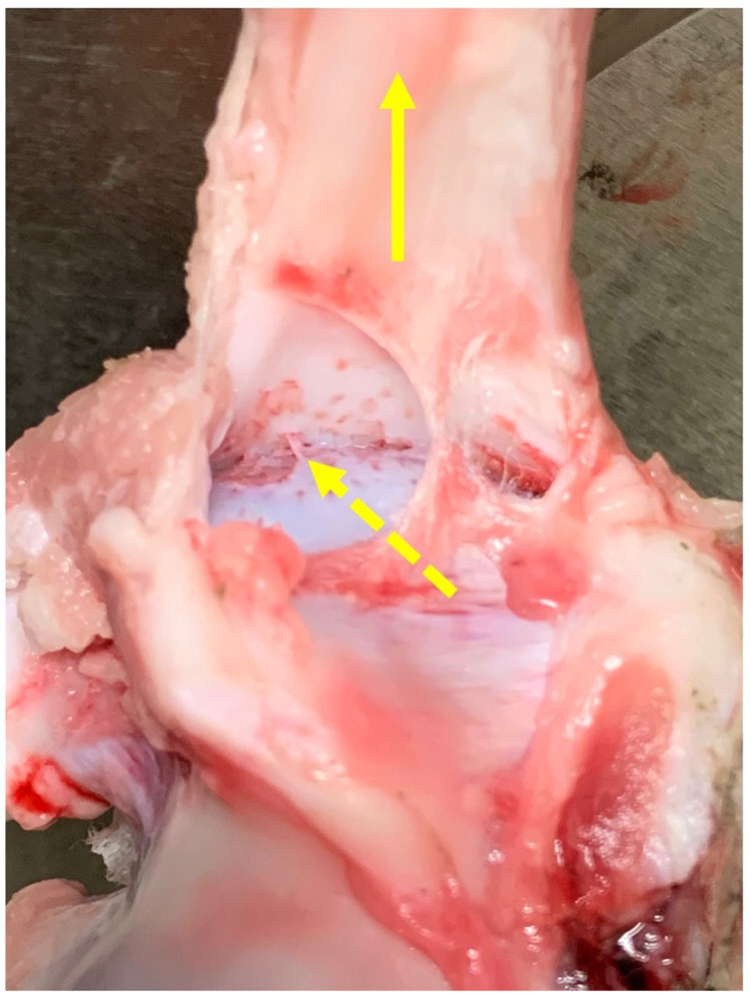
Rotator cuff attachment avulsion at the time of tensile testing. Yellow arrow indicates the direction of the traction force, yellow dotted arrow indicates the site of avulsion between the rotator cuff and the native attachment of the humeral head.

**Figure 3:**
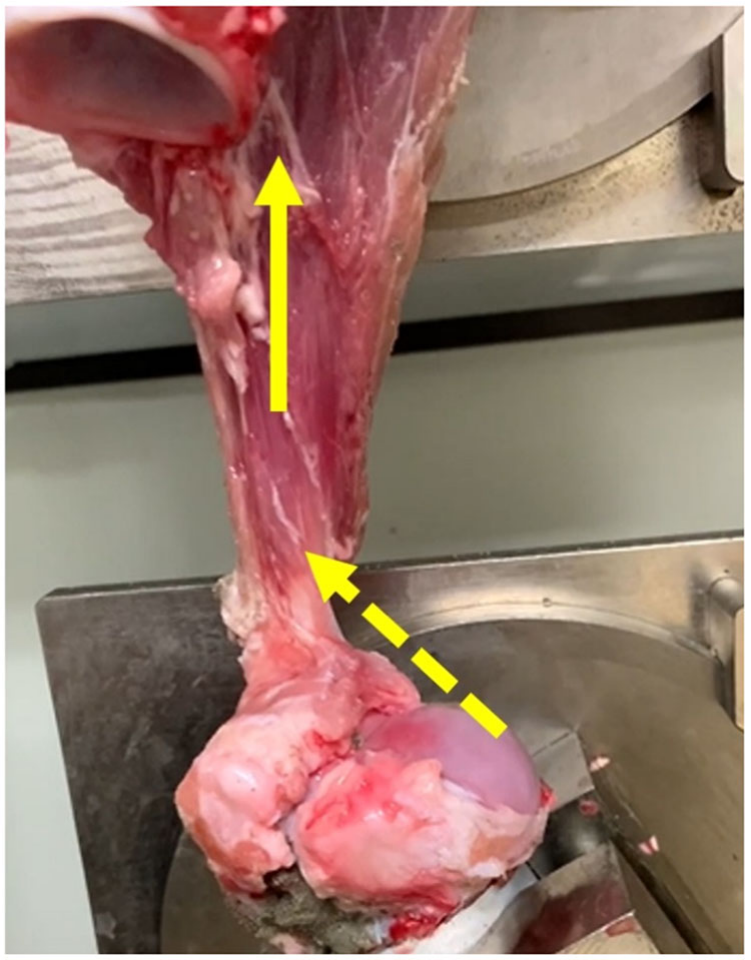
Rupture by pulling at the rotator cuff–tendon transition at the time of tensile testing. Yellow arrow indicates the direction of the traction force; yellow dotted arrow indicates the site of pulling out at the rotator cuff–tendon transition.

Totally 16 fresh porcine cadaver shoulders were evaluated according to the previous study to evaluate the onset of rigour mortis [12]. Nine shoulders (56%) were torn as a result of rotator cuff attachment avulsion, and the remaining seven (44%) were ruptured by pulling at the rotator cuff–tendon transition (Figures 2 and 3). Therefore, we determined that these numbers of specimens per group would provide a power of 80% to detect a difference (*α* < 0.05) with effect size of 1.5 for parametric analysis and 1.6 for nonparametric analysis. The normality of each structural property was evaluated using the Shapiro–Wilk test. Considering the results of the normality test, Student's *t* test or the Mann–Whitney U‐test was used to compare whether the structural properties were different for different failure modes. Results for normally distributed data were reported as mean (standard deviation; SD), whereas results for nonnormally distributed data were presented as median ([first and third interquartile range). EZR [9] was used for statistical tests with a significance level of *p* < 0.05.

## RESULTS

**Figure 4:**
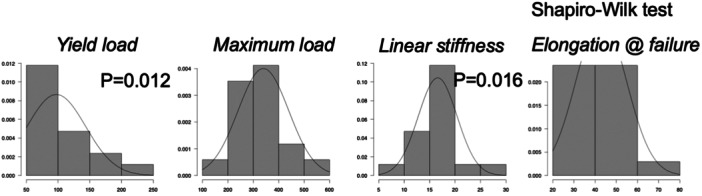
Results of the distributions from the Shapiro–Wilk test for the yield load, maximum load, linear stiffness and elongation at failure.

**Figure 5:**
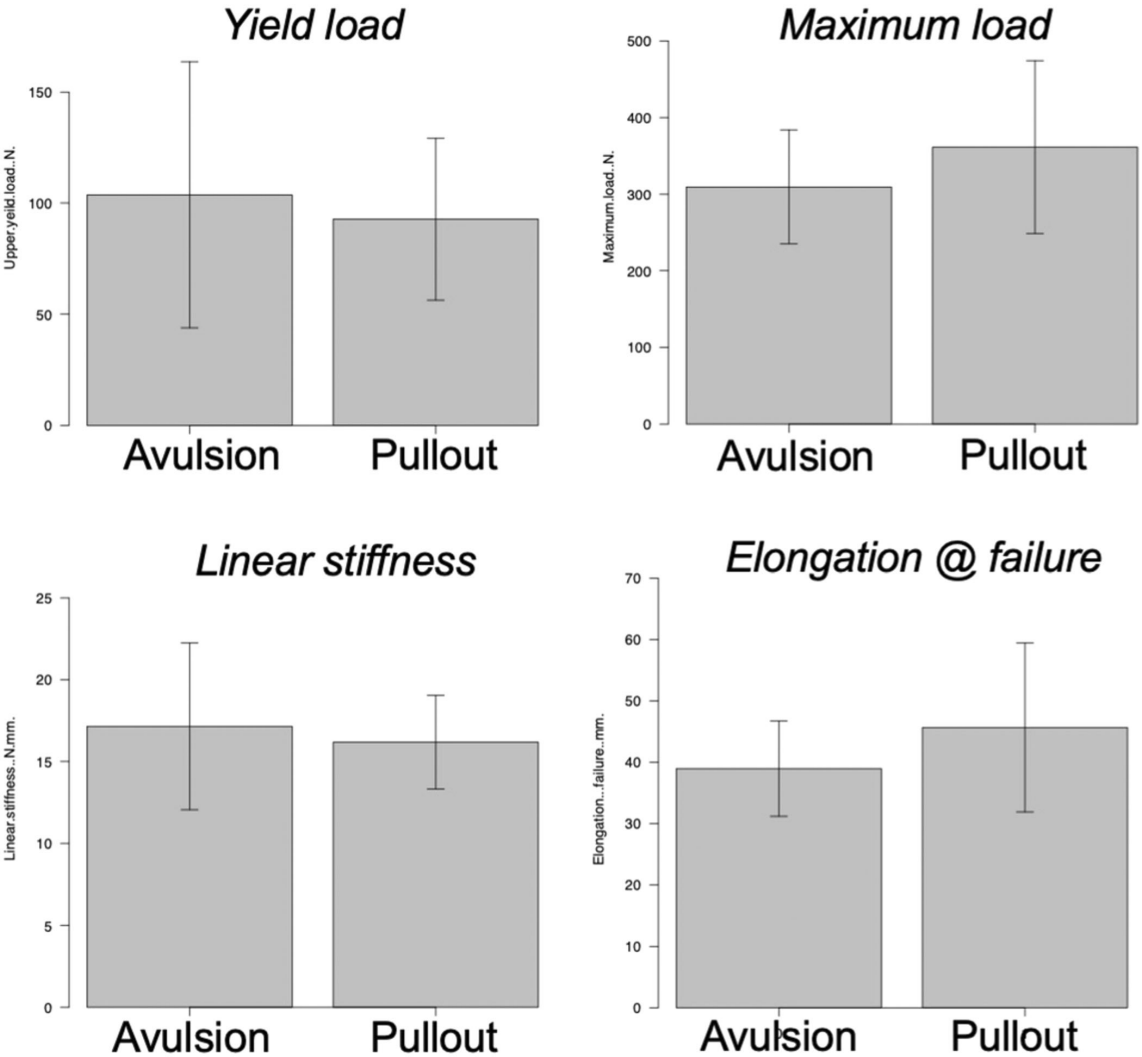
No significant differences in structural characteristics were noted between attachment avulsion and pulling at the rotator cuff–tendon transition.

The maximum load and elongation at failure were normally distributed and were 346.4 (99.2) N and 43.1 (12.2) mm, respectively. The yield load (*p *= 0.016) and linear stiffness (*p *= 0.041) were not normally distributed and were 81.2 (63.0, 121.6) N and 15.8 (14.8, 17.5) N/mm, respectively (Figure 4). No significant differences in structural characteristics were found between attachment avulsion and pulling at the rotator cuff–tendon transition (Figure 5). A summary of the results is shown in Table 1.

**Table 1:** Results of structural properties in tensile testing.

Parameters	Mean (SD)	Median (first and third IQR)	ND	*p* Value
Upper yield load, *N*	99.7 (46.5)	81.2 (63.0, 121.6)	No	0.016
Maximum load, *N*	346.4 (99.2)	345.4 (276.3, 397.0)	Yes	>0.05
Linear stiffness, *N*/mm	16.5 (3.9)	15.8 (14.8, 17.5)	No	0.041
Elongation at failure, mm	43.1 (12.2)	43.4 (34.4, 45.7)	Yes	>0.05

*Note*: The normality of each structural property was evaluated using the Shapiro–Wilk test.

Abbreviations: IQR, interquartile range; ND, normal distribution; SD, standard deviation.

## DISCUSSION

The most important finding of this study was that no significant differences in structural characteristics were noted between attachment avulsion and pulling at the rotator cuff–tendon transition. The model's maximum load did not necessarily reflect the strength of the rotator cuff attachment, and the failure mode did not affect the structural properties. Furthermore, the yield load and linear stiffness were not normally distributed. These results demonstrated the importance of the yield load and failure mode in the preoperative and postoperative biomechanical evaluations of large‐animal rotator cuff injury models. Other evaluation parameters such as cyclic displacement under the force below the yield load should also be considered to evaluate the quality of the repaired rotator cuff postoperatively. To evaluate the structural properties of rotator cuff attachments during mechanical testing, the cyclic load should be determined by percentile rather than by the mean and SD. The structure of porcine rotator cuff attachment is similar to that of sheep and dogs that is commonly used for large animal joint biomechanical studies [2, 18]. Several biomechanical studies using sheep tendon rupture models were reported [3, 6, 17, 19]. However, these studies differ in that a mechanical testing apparatus was applied at the location of the potted humerus, and the tendon was fixed with a cryoclamp. Wang et al. compared the biomechanical properties of rotator cuff repair. Repaired porcine subscapularis tendon underwent failure testing. Failure modes, load to create a 3‐mm gap, failure load and stiffness were compared [20]. Following rotator cuff repair, the humerus was attached to the tester and positioned for vertical loading. On the other hand, that study differed from the present study in that the infraspinatus was removed from the scapula and grasped to apply traction force. It is not possible to simultaneously observe both the tendon attachment and the myotendinous junction during cyclic and failure tests in those settings. On the other hand, Ng et al. performed a controlled laboratory study to assess how a double‐row rotator cuff repair using three to four different configurations of suture anchors affects the area of contact between the rotator cuff–tendon and bone [13]. Porsche et al. evaluated the tendon mobility of fresh porcine cadaver shoulders with artificial rotator cuff tears using the sensor‐enhanced, arthroscopic grasper [14]. In addition, they also analyzed the effect of the interval slide procedure in a fresh porcine cadaver model. They evaluated the changes in tendon mobility through the interval slide procedure and compared them to native tendons [16]. Furthermore, they also evaluated the effect of interval slide procedures on repairing tension using human cadaveric shoulders. These studies were consistent with the present study in that the supraspinatus tendon was not removed from the myotendinous junction [15]. To clarify the biological augmentation to the graft, Hee et al. conducted biomechanical evaluations using an ovine model of rotator cuff repair. They performed acute infraspinatus tendon detachment and repair. After euthanasia and specimen preparation, the humerus was potted in polyvinylchloride pipe using high‐strength polymethylmethacrylate. Traction force was applied using a custom‐designed brass cryoclamp. However, the study was conducted to detect a 50% difference in the ultimate force at failure between the groups [6]. Easley et al. conducted a biomechanical evaluation using an ovine rotator cuff repair model. They performed acute infraspinatus tendon detachment repair using an innovative anchor, a poly (lactic acid‐glycolic acid) scaffold device or a similar anchor without a scaffold. Cryoclamps were also used to grasp the infraspinatus tendon, although load‐to‐failure testing was also assessed in this study and the site of failure during the study was not described [3].

Achilles tendon attachment is similar to the rotator cuff attachment, which is the attachment between the scapula–supraspinatus–tendon complex and the humerus, in that it is a complex of muscle–tendon junction, tendon and attachment to the calcaneus. Several biomechanical studies using repeated testing have been reported to assess Achilles tendon attachment behaviour and tendon stiffness [1, 11]. The present results will help fill in the literature gap on stability after rotator cuff tear repair and are valuable for further clinical studies by an innovative setup including myotendinous junction and scapula. The evaluation of structural properties of the humerus–tendon–scapula complex and the assessment of failure sites is one of the novelties of this study to establish an innovative setup including not only the supraspinatus tendon but also the myotendinous junction and scapula. Hatta et al. investigated the alteration of passive stiffness in the supraspinatus muscle after double‐row and knotless transosseous‐equivalent repair techniques, using shear wave elastography in human cadaver shoulders with rotator cuff tears [5]. This study was similar to the present study in that the musculotendinous junction and scapula were not resected during surgery and experimentation. However, the nature of cadaveric studies in humans does not allow the postoperative stiffness of the repair site to be assessed, and in vivo large animal studies are needed to clarify this.

From the results of the present study, the maximum load cannot always reflect the structural properties of the repaired rotator cuff attachment, and midsubstance tissue failure was commonly observed. Thus, we must consider the location where yielding and rupture occur during tensile testing; if these occur at the rotator cuff musculotendinous junction, both the yield and maximum loads do not reflect the condition of the rotator cuff attachment.

### Limitations

This study has several limitations. First, the study used porcine models; therefore, some findings may not directly translate to clinical practice in humans. Differences between the young human shoulder and the porcine shoulder with respect to trabecular bone characteristics could have affected fracture loads and other parameters. However, porcine shoulders were reported to be similar to human shoulders in many aspects [18]. Second, this study evaluated only the time‐zero structural properties of the scapulohumeral complex, so it was not possible to report the effects on flexion–extension movements and any biological healing response. Because only tensile forces involving isolated portions of the shoulder were tested in vitro, they may not reflect the actual traction forces to which the fixation construct is subjected in vivo. Third, tensile tests were performed in a completely open environment, which does not replicate the original environment. Fourth, given the limited availability of specimens and implants, each group included few samples.

## CONCLUSIONS

Over half of the porcine scapulohumeral complex was torn as a result of rotator cuff attachment avulsion in the load‐to‐failure test. The model's maximum load did not necessarily reflect the strength of the rotator cuff attachment, and the failure mode did not affect any structural properties.

## AUTHOR CONTRIBUTIONS

The conception and design of this study were performed by Tuneari Takahashi and Hideyuki Sasanuma. The acquisition of data was done by Tuneari Takahashi and Hideyuki Sasanuma. Analysis and/or interpretation of data was carried out by Tuneari Takahashi and Hideyuki Sasanuma. The drafting of the article was done by Tsuneari Takahashi, Hideyuki Sasanuma and Katsushi Takeshita. All authors have contributed significantly to the study, approved the article and agreed with the submission.

## CONFLICT OF INTEREST STATEMENT

The authors declare no conflicts of interest.

## ETHICS STATEMENT

Ethical approval was not required due to ex vivo nature of this study. This article does not contain any studies involving human subjects.

## Data Availability

Data and materials of this study are available from the corresponding author upon reasonable request.
